# Design of non-viral vector with improved regulatory features towards therapeutic application

**DOI:** 10.6026/97320630016307

**Published:** 2020-04-30

**Authors:** B.Sharan Sharma, Vaishna Prabhakaran, Ramtej J Verma

**Affiliations:** 1 Indrashil Institute of Science and Technology (IIST)/Indrashil University (IU), Kadi, Mehsana-382740, Gujarat, India; 2 Department of Zoology, Biomedical Technology and Human Genetics, University School of Sciences, Gujarat University, Navrangpura, Ahmedabad-380009, Gujarat, India

**Keywords:** DES-LCR, Microdystrophin, DMD, Non-viral vector, Gene therapy

## Abstract

Viral vectors based gene therapy is often compromised by adverse immunological reactions raising safety concerns. Hence, improved design and development of non-viral vectors with strong regulatory regions is desired. We describe the design of a non-viral mammalian expression vector in which the primary transgene (a truncated dystrophin gene linked with Duchenne muscular dystrophy (DMD)) named microdystrophin delR4-R23/delCT (MD1) is under the transcriptional control of elements of desmin locus control region (DES-LCR). The designed vector, named as DES-LCR/MD1-EGFP, was constructed by cloning two fragments into the pBluescript backbone. Fragment 1 contains DES-LCR enhancer and DES-LCR promoter region while fragment 2 contains MD1 transgene and reporter EGFP (enhanced green fluorescent protein) gene separated by linker P2A (2A peptide). This vector design provides a framework for strong regulation with non-viral features. This design forms the foundation for application in conditions linked to multisystem diseases.

## Background

Applications of gene therapy are tremendous and offers hope to treat genetic diseases at large. Success of gene therapy lies on the design, development and delivery of vectors, which can be of viral and non-viral types. Both, viral and non-viral gene therapy vectors, have applications in preclinical and clinical settings. Viral vectors have emerged as effective gene therapy vehicles for clinical gene therapy [1], however, safety has been an issue on use of viral vectors since they may generate strong immune response [2]. On the other hand, non-viral vectors have been ignored in the past but they certainly represent the long-term future of gene therapy owing to their increased safety. In recent years, interest towards development of non-viral DNA vectors has progressed steadily [3], and several non-viral vector systems have been developed and successfully employed for safe delivery [4]. There are opportunities for further development of non-viral vectors with improved design to address therapeutic needs. One of the solutions to improve the design includes inclusion of strong regulatory features to achieve desired expression of transgenes.

Role of cis-regulatory elements, as strong regulatory sequences, in the design of gene therapy vectors has long been known [5-6]. Sequences of locus control regions (LCRs), non-coding cis-regulatory regions, have been used in the past in viral vectors to control expression of transgenes [7-8]. Ability to control gene expression at ectopic sites makes LCRs unique and different from other distal elements of the genome. Unique regulatory signatures have been identified in the human LCR sequences [9], and further efforts to use these special regulatory elements in the design of gene therapy vectors will pave the way to generate new generation of gene therapy vectors with increased safety.

It is of interest to design a non-viral mammalian expression vector for the expression of a truncated version of dystrophin gene, microdystrophin delR4-R23/delCT (MD1). Typically, truncated versions of dystrophin gene are preferred choice for gene therapy of DMD [10]. Despite the promise of gene therapy for the treatment of DMD, it has been challenging to achieve optimum and prevalent expression of therapeutic gene [11]. Hence, we describe the design of a non-viral vector with improved regulatory features using computer aided sequence analysis tools.

## Methodology:

### Vector design:

SnapGene v5.0 software (from GSL Biotech; available at snapgene.com) was used to rationally design a non-viral mammalian expression vector containing elements of human LCR (DES-LCR) for expression of transgenes (MD1 and EGFP). pBluescript plasmid (3931bp) (adapted from Addgene plasmid #24595) was used as empty backbone to construct the vector. Features of this empty backbone are listed in (Table 1).

### Restriction and insertion cloning of fragments into empty backbone:

Two fragments were cloned into the empty backbone of pBluescript plasmid at different sites using restriction and insertion cloning feature of SnapGene. Fragment 1 (1069bp), containing sequences of DES-LCR enhancer region and DES-LCR promoter region, was cloned at the ApaI (21) and Hind III (257) restriction sites. DES-LCR enhancer (758bp) and promoter (311bp) sequences were retrieved from NCBI (accession numbers NG_046330.1 (17788-18539) and NG_046330.1 (18540-18844) respectively). ApaI and Hind III restriction sequences were added into the 5' and 3' ends respectively of fragment 1 before cloning.

Fragment 2 (4396bp), containing sequences of microdystrophin delR4-R23/delCT (MD1) gene obtained from Foster et al,[12], linker P2A (2A peptide), and reporter EGFP (enhanced green fluorescent protein) gene, was cloned at the EcoRV (265) and BsaBI (826) restriction sites. MD1 is a truncated version (3612bp) of the dystrophin gene used for gene therapy of Duchenne muscular dystrophy (DMD). MD1 sequence was retrieved from Foster et al, [12] which is a codon optimized sequence for maximal expression. P2A (57bp) and EGFP (727bp) sequences were taken from Addgene plasmid #111814. EcoRV (265) and BsaBI (826) restriction sequences were added into the 5' and 3' ends respectively of fragment 1 before cloning. Constructed vector (with cloned fragments) was named as DES-LCR/MD1-EGFP.

### Amplification of MD1 gene:

From the constructed DES-LCR/MD1-EGFP vector sequence, forward and reverse primers, MD1 F (24bp) and MD1 R (29bp) respectively, were designed using the ‘add primer’ option of SnapGene for the in-silico amplification of MD1 gene. Restriction sites were added in the primers for future cloning of the MD1 gene. MD1 F contain EcoRV restriction site and MD1 R contain HindIII restriction site (Table 2). 'PCR' feature of SnapGene was used to amplify the MD1 gene from the constructed DES-LCR/MD1-EGFP vector. MD1 F and MD1 R primers were selected and in-silico PCR was run. Further, 'simulate agarose gel' option was used to confirm the size of amplified MD1 gene on 1% agarose.

### Translation of transgenes:

MD1 and EGFP transgenes were translated to generate amino acid products using the 'translation feature' of SnapGene and HindIII are also present at the start and end, respectively, of the amplified sequence for future cloning purposes.

## Results

### Designed DES-LCR/MD1-EGFP vector:

Non-viral mammalian expression vector, constructed by cloning two different fragments into the pBluescript backbone using SnapGene, was named as 'DES-LCR/MD1-EGFP' vector. DES-LCR/MD1-EGFP is an 8588bp long circular vector characteristics of which are given in (Table 3). DES-LCR/MD1-EGFP is a high copy number vector for growth in bacteria and contains Ampicillin resistance gene, which confers resistance to antibiotic Ampicillin for selection during bacterial growth. Polyadenylation signal from SV40 poly(A) sequence of the vector helps in addition of poly(A) tail to generated mRNAs from the transgenes during propagation in mammalian cells.

### Cloned fragments in the DES-LCR/MD1-EGFP vector:

**Figure 1:**
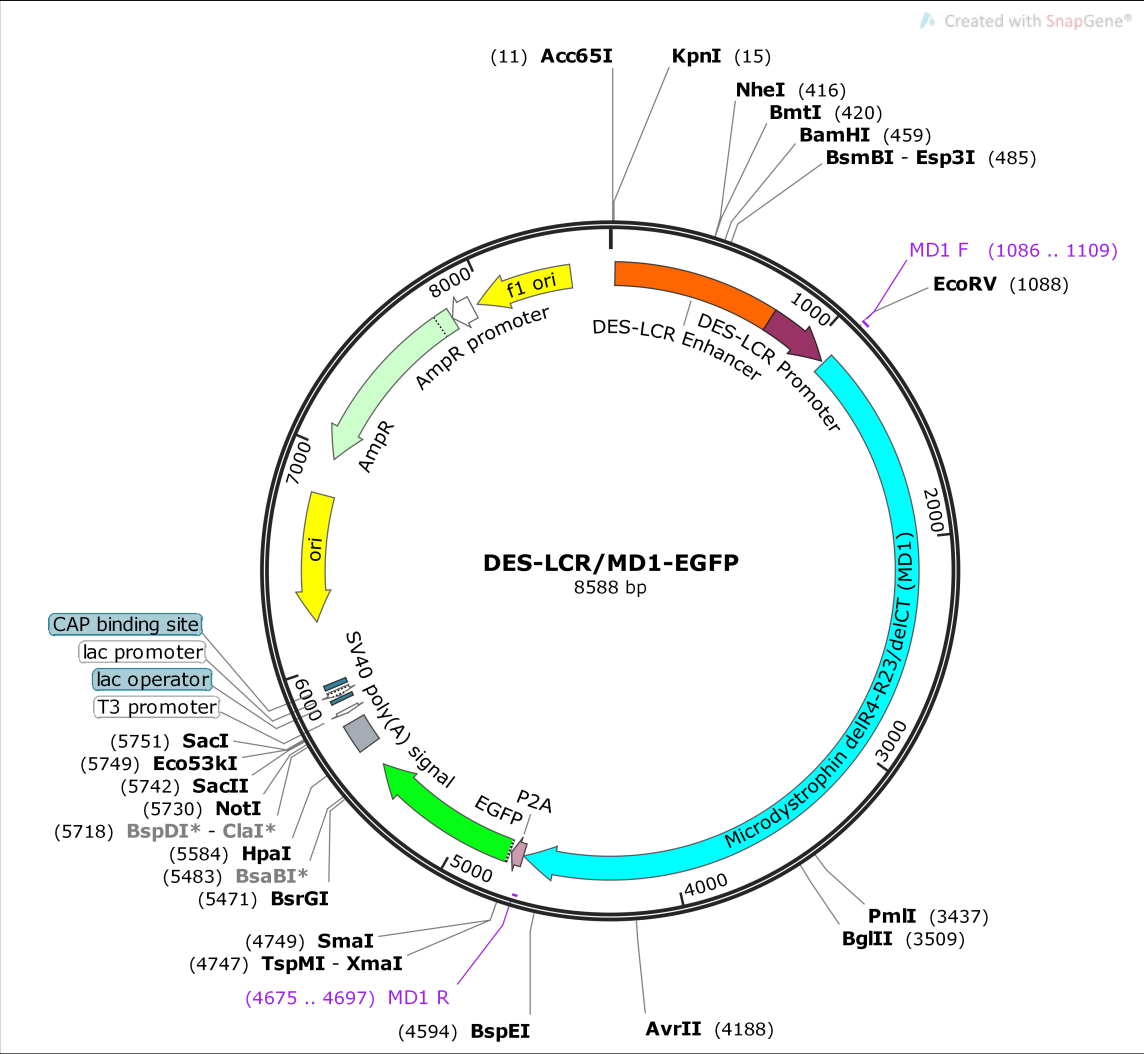
Map of constructed DES-LCR/MD1-EGFP vector. The vector displays the cloned fragments in the pBluescript backbone. Cloned regions are from locations 23 to 1079 (fragment 1: DES-LCR Enhancer and DES-LCR Promoter) and 1092 to 5478 (fragment 2: MD1, P2A and EGFP). Primer binding regions to amplify the MD1 transgene are from locations 1086 to 1109 (MD1 F) and 4675 to 4697 (MD1 R).

Fragment 1 in the vector is located form position 23 to 1079 and fragment 2 is located from position 1092 to 5478. Components of fragment 1, DES-LCR enhancer and promoter, are located from 23 to 774 and 775 to 1079 respectively (Table 4). Components of fragment 2, MD1 gene, P2A sequence and EGFP gene, are located from 1092 to 4697, 4699 to 4755 and 4762 to 5478, respectively. Map of the vector is given as (Figure 1).

### Amplified MD1 gene:

**Figure 2:**
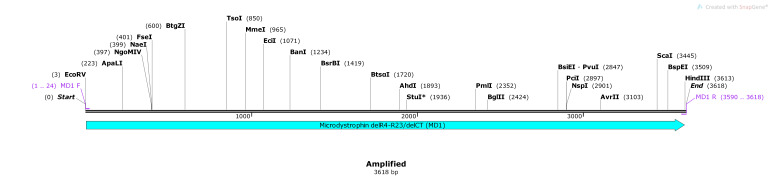
Map of amplified MD1 gene (3618 bp) using MD1 F and MD1 R primers. Map shows the presence of multiple restriction sites within the amplified region. Added restriction sites EcoRV and HindIII are also present at the start and end, respectively, of the amplified sequence for future cloning purposes.

MD1 gene was in-silico amplified using designed MD1 F and MD1 R primer pair. MD1 F (24-mer) and MD1 R (29-mer) primers bind to the 1086 to 1109 and 4675 to 4697 binding sites respectively in the DES-LCR/MD1-EGFP vector with a Tm of 64°C and 60°C respectively. Both these primers contain specific restriction sites for future cloning purposes. Upon in-silico PCR, MD1 F and MD1 R primers generated expected 3.6kb amplicon/product size of which was confirmed by checking the position of amplicon on ‘simulated agarose gel' image. Map of amplified MD1 gene highlighting presence of various restriction sites is given as (Figure 2).

### Translated transgenes:

Upon translation, open reading frames (ORFs) of the transgenes MD1 (3594bp) and EGFP (717bp) generated 1197aa (137.8kDa) and 239aa (26.9kDa) long products, respectively.

## Discussion:

With viral vectors dominating cell and gene therapy, non-viral vectors sidestep the main concerns that come with using viruses: safety, immunogenicity and manufacturing limits (yield, scaling-up and costs) [13]. New generation of vectors with increased safety are desirable for gene therapy of constitutional disorders to achieve permanent genetic modification and stable expression of transgenes. Introduction of novel and strong regulatory elements in the non-viral vectors provides a solution towards improved design of gene therapy vectors.

LCRs are unique non-coding regulatory sequences with their ability to control gene expression at ectopic locations. These regulatory sequences have not been studied much in the past, however, their presence in the mammalian genomes makes them important. LCRs have the ability to enhance the expression of linked genes to physiological levels indicating that they play a significant role in controlling the expression of target genes [14].Potential of using LCR elements in vector design for expression of transgenes have been realized in the past [7-8,15]. However, majority of them are viral vectors. In this work, we designed a non-viral mammalian expression vector via computer-aided tools expressing transgenes under control of elements of LCR.

Despite the availability of gene therapy for the treatment of DMD, many current challenges associated with the therapy are yet to be overcome. Hence, improved next-generation vectors to overcome some challenges of gene therapy for gene diseases like muscle disorders are urgently needed. Computational tools have proven to be very powerful in the systematic and rational design and analysis of vectors of users' choice [16,17]. Recently, Sarcar et al, [18] reported muscle-directed gene therapy by in silico vector design. They used AAV vector in combination with novel cis-regulatory modules (CRMs), containing clusters of TFBSs, to substantially increase muscle-specific gene transcription. However, use of viral vector may limit the success of designed vector.

Constructed DES-LCR/MD1-EGFP vector contains transgene MD1, a codon optimized and truncated version of dystrophin gene [12], under expression control of DES-LCR elements. Codon optimization by synonymous substitution is key to enhanced expression of recombinant protein in host cells [19]. P2A sequence in the vector links the MD1 gene to reporter gene EGFP. P2A is a self-cleaving peptide sequence, which has been extensively used for co-expression of multiple genes at a desired ratio in gene therapy and other biomedical research [20-21]. In silico analysis of DES-LCR/MD1-EGFP vector revealed that this vector could be used to express recombinant genes under control of elements of DES-LCR. It has been reported that enhancer and promoter region sequences of DES-LCR generated high level of expression comparable to highly active constitutive human cytomegalovirus (hCMV) promoter/enhancer [22], suggesting that DES-LCR constitute a promising control region, which can be used in expression vectors. Subsequently, elements of DES-LCR have been used in muscle-directed gene therapy viral vectors [18, 23].

DES-LCR/MD1-EGFP is a non-viral mammalian expression vector and hence a safer option over use of viral vectors expressing dystrophin gene for applications in muscle-directed gene therapy. Functional studies will be needed to validate the performance and usefulness of this vector in therapeutic settings. In recent past, novel designs of non-viral vector systems have been described with promising applications [24]. The designed vector in this work is another step towards tackling, potentially, the current challenges of vector design, which may prove to be useful in future, studies for treating multisystem diseases. Use of non-viral vectors comprising of human elements have been suggested ideal for human gene therapy as they deliver sustainable therapeutic levels of gene expression without adverse immunological effects [25]. Human LCRs are strong regulatory elements, hence, can be considered as preferred choice to construct additional regulatory systems for their applications in biomedical research. Ultimately, these vectors will inflate the traditional applications of gene therapy and will also lead towards newer other opportunities in the field of basic science and clinical research.

## Conclusions:

We describe the design of non-viral vector with improved regulatory features using computer aided sequence analysis tools. This forms a framework towards design of new generation of gene therapy vectors with increased safety. It should be noted that this design should be validated with adequate experimental data.

## Figures and Tables

**Table 1:** Major elements of the pBluescript empty backbone (3931bp)

Element	Location	Size (bp)
SV40 poly(A) signal	928 to 1062	135
T3 promoter	1108 to 1126	19
Lac operator	1171 to 1187	17
Lac promoter	1195 to 1225	31
CAP (catabolite activator protein) binding site	1240 to 1261	22
Ori (Origin of replication)	1549 to 2137	589
AmpR (Ampicillin resistance)	2308 to 3168	861
AmpR promoter	3169 to 3273	105
F1 ori (F1 bacteriophage origin of replication)	3299 to 3751	453

**Table 2:** Designed primers for MD1 gene amplification

Primer	Sequence (5' to 3')	Length	Added Restriction Site
MD1 F	GATATCGCCACCATGCTGTGGTGG	24-mer	EcoRV
MD1 R	AAGCTTTCATCATCACATGGTGTCGGTCT	29-mer	HindIII

**Table 3:** Characteristics of the designed DES-LCR/MD1-EGFP vector (8588bp)

BACKBONE	
Vector backbone	pBluescript
Vector type	Mammalian expression
GROWTH IN BACTERIA	
Bacterial resistance	Ampicillin
Growth temperature	37 ° C
Copy number	High Copy
INSERT	
Fragment 1	DES-LCR enhancer and promoter sequences
Fragment 2	MD1 and EGFP genes separated by P2A sequence
Species	Homo sapiens
Cloning method	Restriction and insertion (SnapGene)

**Table 4:** Location of cloned fragments in the constructed DES-LCR/MD1-EGFP vector

Fragment	Total Size	Components	Size (bp)	Location
Fragment 1	1069bp	DES-LCR Enhancer	758	23 to 774
		DES-LCR Promoter	311	775 to 1079
		Microdystrophin delR4-R23/delCT (MD1)	3612	1092 to 4697
Fragment 2	4396bp	P2A (2A peptide)	57	4699 to 4755
		EGFP (Enhanced Green Fluorescent Protein)	727	4762 to 5478
